# Tumor Tropic Delivery of Hyaluronic Acid-Poly (D,L-lactide-co-glycolide) Polymeric Micelles Using Mesenchymal Stem Cells for Glioma Therapy

**DOI:** 10.3390/molecules27082419

**Published:** 2022-04-08

**Authors:** Xiao-Ling Wang, Wen-Zheng Zhao, Jia-Ze Fan, Le-Chen Jia, Ya-Nan Lu, Ling-Hui Zeng, Yuan-Yuan Lv, Xiao-Yi Sun

**Affiliations:** 1 Department of Pharmacy, Zhejiang University City College, Hangzhou 310015, China; lingxiaowangjy@163.com (X.-L.W.); 18742525327@163.com (W.-Z.Z.); fxh23205549@163.com (J.-Z.F.); kfjialechen@163.com (L.-C.J.); luyanan991030@163.com (Y.-N.L.); zenglh@zucc.edu.cn (L.-H.Z.); lvyy@zucc.edu.cn (Y.-Y.L.); 2 Department of Pharmacy, School of Ophthalmology & Optometry and Eye Hospital, Wenzhou Medical University, Wenzhou 325035, China; 3 Institute of Pharmaceutics, College of Pharmaceutical Sciences, Zhejiang University, Hangzhou 310058, China

**Keywords:** mesenchymal stem cells, hyaluronic acid, poly (lactide-co-glycolide), orthotopic glioma, tumor penetration

## Abstract

Tumor penetration and the accumulation of nanomedicines are crucial challenges in solid tumor therapy. By taking advantage of the MSC tumor-tropic property, we developed a mesenchymal stem cell (MSC)-based drug delivery system in which paclitaxel (PTX)-encapsulating hyaluronic acid-poly (D,L-lactide-co-glycolide) polymeric micelles (PTX/HA-PLGA micelles) were loaded for glioma therapy. The results indicated that CD44 overexpressed on the surface of both MSCs and tumor cells not only improved PTX/HA-PLGA micelle loading in MSCs, but also promoted the drug transfer between MSCs and adjacent cancer cells. It was hypothesized that CD44-mediated transcytosis played a crucial role and allowed deep glioma penetration depending on sequential intra–intercellular delivery via endocytosis–exocytosis. MSC-micelles were able to infiltrate from normal brain parenchyma towards contralateral tumors and led to the eradication of glioma. The survival of orthotopic glioma-bearing rats was significantly extended. In conclusion, the MSC-based delivery of HA-PLGA micelles is a potential strategy for tumor-targeting drug delivery.

## 1. Introduction

Tumor penetration by nanomedicines is a crucial challenge in solid tumor therapy. Elevated interstitial fluid pressure (IFP) and the high viscosity of the extracellular matrix (ECM) hindering the diffusion of nanomedicines compromise the effectiveness of antitumor therapy. Strategies including particle size and geometry optimization [1], ECM viscosity modulating [2], tumor-penetration peptide modification [3], and tumor microenvironment-responsive carrier design [4], have emerged to facilitate the movement of nanoparticles. Transcellular and paracellular pathways participate in tumor penetration across multiple cell layers. Recently, the important role of transcytosis concerned in the active transcellular transportation process that happens in the endothelia cells and cancer cells in tumor penetration has been underlined [5,6]. The intratumor penetration relying on sequential intra–intercellular delivery via endocytosis–exocytosis is favorable to circumvent the hindrance of ECM and provide a driving force for penetration [7]. Nanoparticles are reported to be excreted from cancer cells after internalization. The exocytosed particles then infect adjacent cells, allowing the delivery of anticancer drugs deep into avascular tumor tissue. Inspired by the improved efficacy of receptor-mediated transcytosis across the endothelium, pervasive tumor penetration has been achieved by hyaluronic acid- (HA) [8,9] and folic acid-modified nanoparticles [6]. The interaction between ligand and receptor not only promotes the endocytosis, but also boosts cargo recycling back to the outside.

Mesenchymal stem cells (MSCs) are multipotent cells endowed with homing, immunomodulating, and differentiation activities. Chemokine attraction in the tumor microenvironment, endothelial adhesion and transmigration lead to MSC recruitment and engraftment into tumorigenic sites [10,11]. Therefore, MSCs have emerged as one of the most promising choices in cell-based drug delivery to improve tumor targeting. Besides endothelia and cancer cells, the phenomenon of payload excretion is also common in stem cells. Therefore, MSC-hitchhiking with genes, chemotherapeutic drugs, nanoparticles, and theranostic agents have been explored to enhance control over the distribution of drugs and improve targeting in antitumor therapy [12,13]. In our previous investigation, MSC-mediated paclitaxel-poly (D,L-lactide-co-glycolide) nanoparticle (PTX/PLGA NPs) delivery was successfully established for glioma therapy [14]. MSCs were able to off-load PTX by NP exocytosis in a sustained and efficient manner. The antitumor activity is expected to be potentially improved if more PTX could be released from MSCs and actively infiltrate throughout the glioma to reach the distal cells.

In regard to the overexpressed CD44 in both MSCs and tumor cells [15], MSCs are supposed to load more nanoparticles with HA coating through receptor-mediated endocytosis. When CD44 is recycled to the cell surface from the endosomes, it can bring the bound HA species to the surface and release them to the extracellular space [16]. As a result, the enhanced transcytosis is also achieved. Herein, with CD44 recycling between the cell surface and interior of tumor cells, an MSC-based “Trojan horse” loaded with PTX/HA-PLGA polymeric micelles (MSC-micelles) is fabricated to gain deeper tumor penetration and more active-targeting for orthotopic glioma therapy to overcome non-specific distribution attributed to the wide expression of CD44 on endothelial cells, epithelial cells, fibroblasts, keratinocytes, and leukocytes delivered by PTX/HA-PLGA micelles [17]. MSC-micelles exhibited potent tumor tropism, efficient drug transfer to tumor cells, and strong anti-glioma effects with minimal adverse effects. This study offers an excellent nano-platform for engineering tumor-targeting drug delivery systems.

## 2. Results

### 2.1. Characterization of HA-PLGA and PTX/HA-PLGA Micelles

The molecular weights of PLGA and HA-PLGA determined by GPC were 19,480 g/mol and 23,851 g/mol, respectively. The increment 4371 g/mol was in accordance with the molecular weight of HA (5763 g/mol). Additionally, based on the ^1^H-NMR results and critical micelle concentration (CMC) value we reported previously [18], HA-PLGA was successfully synthesized.

The particle size of the PTX/HA-PLGA micelles was 141.2 ± 0.5 nm with a narrow size distribution (PDI 0.055 ± 0.08). The micelles exhibited a negative charge (−16.6 ± 0.4 mV) in PBS (pH 7.4) due to the HA chain. The micelles were physically stable at 4 °C for 3 weeks, with slightly changes in size (130.2 ± 0.7 nm) and zeta potential (−15.1 ± 0.1 mV). There was no significant sign of drug leakage occurring in the storage period with the drug loading rate (2.57 ± 0.03% vs. 2.27 ± 0.05%) and encapsulating efficiency (53.64 ± 1.08% vs. 51.78 ± 1.11%) before and after stability test, respectively. As the control particle, the PTX/PLGA NP was 133.7 nm ± 2.8 nm with zeta potential of −25.6 ± 3.1 mV. The loading rate was 4.12 ± 0.23%.

**Figure 1:**
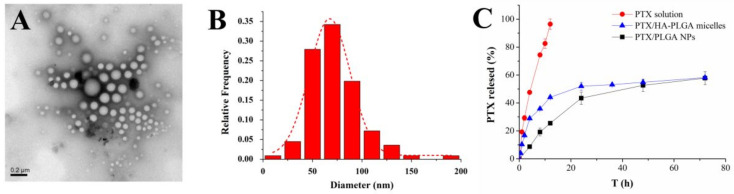
(**A**) TEM image of PTX/HA-PLGA micelles. (**B**) Particle size distribution curve based on TEM results. (**C**) In vitro release of PTX/HA-PLGA micelles, PTX/PLGA NPs, and PTX solution.

The transmission electron microscopy (TEM) image presented spherical-shaped and well-dispersed PTX/HA-PLGA micelles (Figure 1A,B). Because the TEM samples were observed in vacuum in a dry state, the average size for TEM was much smaller than that for DLS. PTX/HA-PLGA micelles and PTX/PLGA NPs showed the similar biphasic drug release pattern (Figure 1B). In the first 24 h, a burst release appeared with 40% PTX release, followed by a sustained release in another 48 h. About 60% PTX could be finally released within 3 days.

### 2.2. Endocytosis Pathways and Cytotoxicity of PTX/HA-PLGA Micelles

**Figure 2:**
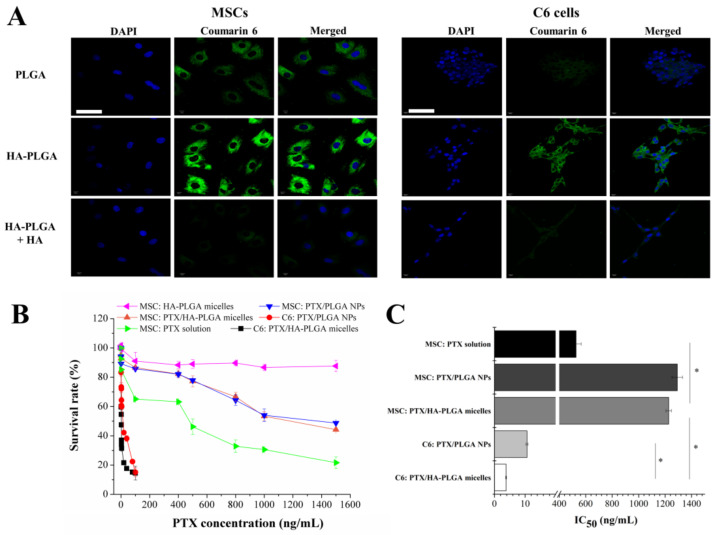
(**A**) Uptake of coumarin 6/PLGA NPs, coumarin 6/HA-PLGA micelles and coumarin 6/HA-PLGA micelles combined with free HA. The nucleus was stained with DAPI (blue) and green represented coumarin 6 (bar = 50 µm). (**B**) Cytotoxicity of PTX/HA-PLGA micelles, PTX/PLGA NPs, and PTX solution against MSCs and C6 glioma cells. Cell viability was determined by MTT assay after incubation with PTX formulations for 72 h. (**C**) IC_50_ values of PTX formulations in MSCs and C6 cells (* *p* < 0.05).

Flow cytometry results demonstrated that both MSCs and C6 cells were positive for CD44 (Figure S1). HA-modified micelles significantly enhanced the cellular uptake via HA-CD44 interaction (Figure 2A). The fluorescence of the coumarin 6/HA-PLGA micelles-treated group was higher than its counterpart (coumarin 6/PLGA NPs). The significantly reduced uptake after competitive treatment with free HA confirmed that HA-PLGA micelles were efficiently internalized by receptor-mediated endocytosis in CD44 over-expressing cells.

The intracellular drug level was closely related to the cytotoxicity. Therefore, PTX-loaded HA-PLGA micelles were more toxic than PLGA NPs against C6 cells (IC_50_ 3.8 ± 0.14 ng/mL vs. 10.67 ± 0.34 ng/mL). Glioma cells were more sensitive to PTX (Figure 2B). A dose-dependent and significant cytotoxicity was observed in C6 cells within 100 ng/mL while MSCs were still alive at a substantially higher level (1500 ng/mL). PTX tolerance enabled MSC to be a viable vehicle for the delivery of the antitumor agent, and resulted in the similar IC_50_ of PTX/HA-PLGA micelles (1226.81 ± 20.53 ng/mL) and PTX/PLGA NPs (1292.23 ± 40.12 ng/mL) against MSCs. Due to the encapsulation of PTX into nanocarriers, MSCs were protected from direct exposure to hazard, while free PTX behaved the most toxically to MSCs (Figure 2C). Blank HA-PLGA micelles were nontoxic to MSCs. Compared with MSC-NPs, MSC-micelles could load more PTX and keep MSCs alive as evidenced by the uptake and cytotoxicity results.

**Figure 3:**
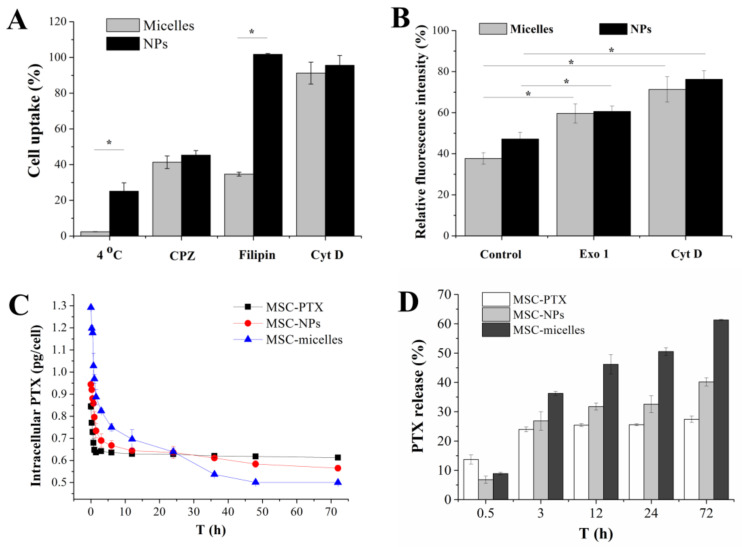
(**A**) Relative uptake of coumarin 6/PLGA NPs and coumarin 6/HA-PLGA micelles in MSCs pre-incubated at 4 °C or with three endocytic inhibitors, 10 µg/mL CPZ, 1 µg/mL filipin III, and 1 µg/mL Cyt D. The intracellular fluorescence intensity of coumarin 6/PLGA NPs and coumarin 6/HA-PLGA micelles in MSCs treated under 4 °C or without endocytic inhibitors was set at 100%, respectively (* *p* < 0.05). (**B**) The effect of Exol 1 and Cyt D on the exocytosis of coumarin 6/PLGA NPs and coumarin 6/HA-PLGA micelles in MSCs (* *p* < 0.05). Cells were incubated with coumarin 6-loaded nanocarriers for 4 h, followed by incubation in fresh medium in the presence or absence of 50 µM Exo 1, 10 µg/mL Cyt D for 2 h, respectively. The intracellular fluorescence intensity of MSCs treated with coumarin 6/PLGA NPs and coumarin 6/HA-PLGA micelles for 4 h was set at 100%, respectively (* *p* < 0.05) (**C**) Release kinetics profiles of PTX from MSC-PTX, MSC-NPs, and MSC-micelles. (**D**) The percentage of PTX released from MSC.

To clarify the energy dependence and internalization pathway mechanisms, specific endocytic inhibitors were used. Namely, MSCs were pre-incubated at 4 °C or with three pharmacological inhibitors: CPZ, filipin, and Cyt D for clathrin-, caveolae-dependent endocytosis, and macropinocytosis, respectively. Lower temperature (4 °C) and CPZ inhibition caused a marked drop in both PLGA NP and HA-PLGA micelle uptake, suggesting the full active energy-dependent and clathrin-mediated endocytic process (Figure 3A). Macropinocytosis inhibitor Cyt D did not cause uptake reduction of either PLGA NPs or HA-PLGA micelles. It is noteworthy that filipin did not affect the uptake of PLGA NPs, while more than 60% uptake of fluorescent micelles were drastically blocked in MSCs. From these results, we inferred that the MSCs’ internalization of PLGA NPs depended on clathrin-mediated endocytosis. Both clathrin- and caveolae-mediated endocytosis were involved in the internalization of HA-PLGA micelles.

### 2.3. Exocytosis of Micelles and PTX Release from MSC-Micelles

Endocytosis and exocytosis represent two different transport processes in opposite directions. Drug release from MSC-micelles depends on the particle exocytosis from MSCs. Golgi network and lysosomes are involved in prominent ways for cell egress of nanomedicines [19]. Exo 1 can disrupt Golgi apparatus or caveosomes and function as a chemical inhibitor of the exocytosis pathway [20]. Cyt D is able to inhibit the polymerization of actin which is required to deliver lysosomes to the cell edge and fuse with cell membrane [21]. Exo 1 and Cyt D significantly decreased the exocytosis of PLGA-based nanoparticulates which in turn increased the cytoplasmic fluorescence in MSCs (Figure 3B), suggesting that both HA-PLGA micelles and PLGA NPs underwent lysosomal exocytosis and Golgi/caveosome excretion (* *p* < 0.05). Exo 1 had a greater impact on the exocytosis of HA-PLGA micelles than PLGA NPs. Relative fluorescence intensity increased more than 20%. Cyt D had a similar inhibition effect (about 30%) on both nanoparticle and micelle excretion.

Drug release kinetics from MSCs was recorded by intracellular PTX determination after incubating MSC-micelles with drug-free medium (Figure 3C). The significantly enhanced accumulation of PTX/HA-PLGA micelles was confirmed by PTX levels at the starting point. MSCs took up about 1.5 times more PTX in the micelles group than those in the PLGA NPs group. Both MSC-PTX and MSC-nanocarriers displayed the initially rapid and then slow release pattern. The drug in MSC-PTX was quickly eliminated in the first 6 h and the residue maintained at a stable level in the following 66 h. In contrast, a sustained intracellular drug reduction, especially MSC-micelles, could be observed in MSC-nanocarriers during the 3 days of the in vitro release test. Finally, about 25, 40 and 60% intracellular PTX corresponding to 0.23 ± 0.02, 0.38 ± 0.02, and 0.79 ± 0.015 pg/cell PTX was released from MSC-PTX, MSC-NPs and MSC-micelles, respectively (Figure 3D). More deliverable drug to the tumor cells was achieved by MSC-hitchhiking with HA-PLGA micelles.

### 2.4. Characterization of MSC-Micelles

**Figure 4:**
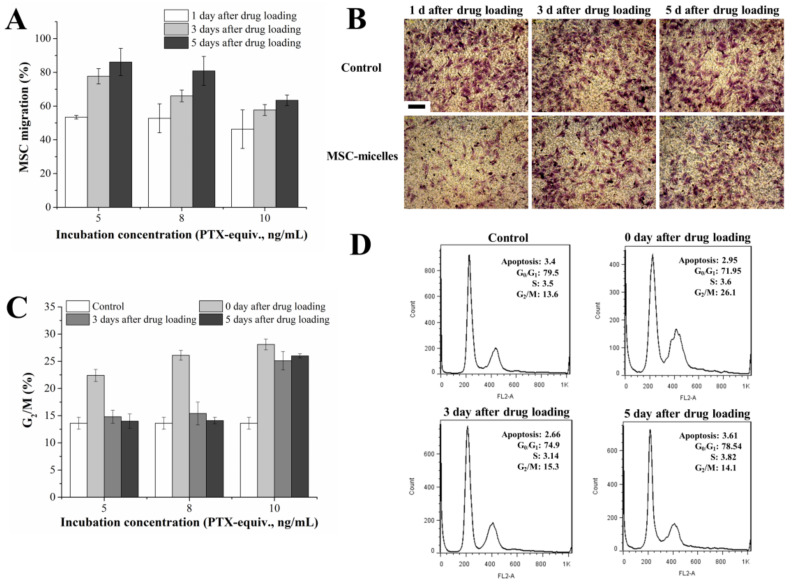
(**A**) Migratory activity of MSC-micelles with different drug loading and recovery times. The numbers of the migrating MSCs were counted. Cells incubated with the blank micelles were set as the control. (**B**) Representative images of migrated MSCs incubated with 8 ng/mL PTX/HA-PLGA micelles through the membrane pores; 1, 3, 5 days after MSC-micelles seeding to the upper chamber of the transwell system, the migrated MSCs were stained by crystal violet (bar = 300 µm). (**C**) The percentage of MSC-micelles arrested in G_2_/M phase 0, 3, 5 days after incubation with 5, 8, 10 ng/mL PTX/HA-PLGA micelles. (**D**) Cell cycle analysis of MSCs recovered from exposure to 8 ng/mL PTX/HA-PLGA micelles.

MSC-micelles were obtained by incubation of MSCs with PTX/HA-PLGA micelles. PTX concentration was a determinant for drug loading and MSC-micelle activity. The tumor tropism of the MSC-micelles was analyzed by a transwell migration test. The results showed that 5–10 ng/mL PTX/HA-PLGA micelle incubation could induce an approximately 50% decrease in the number of migratory MSCs. After cell culture with drug-free medium, MSCs could recover from the migration inhibition in a dose- and time-dependent way. About 80% of MSCs regained their migration capacity 5 days after drug loading both in 5 ng/mL and 8 ng/mL groups (Figure 4A). However, in the high dosage group (10 ng/mL), there was as much as 40% MSCs that could not migrate through the membrane pore on the 5th day. Representative images of migrated MSCs loaded with 8 ng/mL PTX/HA-PLGA micelles are shown in Figure 4B. The results demonstrated almost recovery of MSC-micelles (8 ng/mL) migration capacity 3–5 days after drug treatment.

As an anti-microtubule agent, PTX would arrest MSCs in the G_2_/M phase. The cell cycle pattern of MSCs was monitored for 5 days after micelle loading. Similar to the results of the migration assay, the proportion of MSCs blocked in the G_2_/M phase significantly increased after PTX incubation. Then, a dose- and time-dependent restoration from G_2_/M arrest was observed (Figure 4C). No obvious difference of G2/M percentage was observed either in the low or middle dosage group 3 days and 5 days after drug loading between MSC-micelles and untreated MSCs. In contrast, high drug loading (10 ng/mL) caused continuous G_2_/M phase blocking. In order to achieve high PTX loading, undisturbed cell cycle progression (Figure 4D) and potent migration capacity, MSC-micelles were prepared by incubating MSCs with 8 ng/mL PTX/HA-PLGA micelles for further study.

### 2.5. Drug Transfer in Monolayer and 3D Model of Glioma Cells

**Figure 5:**
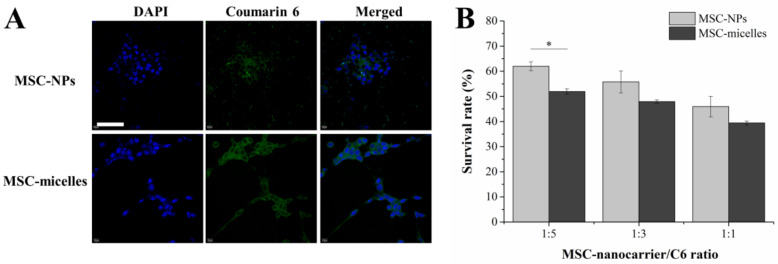
(**A**) CLSM images of C6 cells seeded in the bottom chamber while MSC-nanocarriers loaded with coumarin 6 were seeded in the upper chamber. The nuclei were stained with DAPI (bar = 50 µm). (**B**) Antitumor effects of MSC-micelles against C6 cells with different cell number ratios in the transwell system (* *p* < 0.05).

The transwell system which separated MSC-micelles from C6 cells was used to investigate the drug transfer from MSCs to glioma. From the confocal laser scanning microscopy (CLSM) results in the C6 monolayer, MSC-micelles seeded on the upper chamber could deliver much more fluorescent probe into the tumor cells than the MSC-NPs (Figure 5A). When the ratio of MSC/C6 cell number fixed at 1:5, C6 viability in the MSC-micelles group was significantly lower than that in the MSC-NPs group (Figure 5B). This result could be explained by the fact that the MSC-micelles release more PTX than MSC-NPs as described in the kinetics section. Moreover, the exocytosed PTX/HA-PLGA micelles from MSC-micelles could be endocytosed by C6 cells in a more efficient way than PTX/PLGA NPs because of the HA-CD44 interaction.

**Figure 6:**
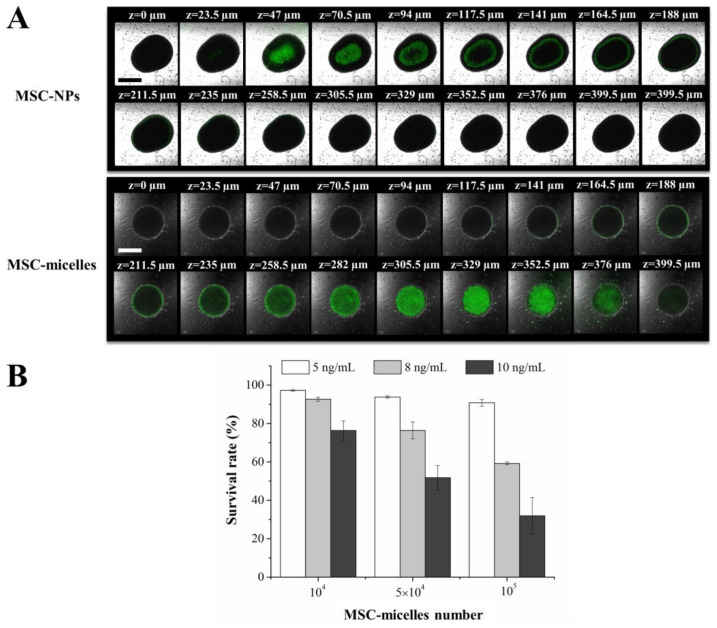
(**A**) Z-stack image of C6 spheroids after incubation with MSC-micelles and MSC-NPs loaded with coumarin 6 (bar = 500 µm). (**B**) Viability of spheroids exposed to MSC-micelles and MSC-NPs loaded with PTX at different drug loading and MSC number.

To overcome the deviations from the evaluation in a 2D monolayer, we used the 3D tumor spheroid model to simulate the essential properties of solid tumor in vitro. Herein, the penetration of MSC-micelles loaded with coumarin 6 was observed, the antitumor effect of PTX-loaded MSC-micelles on tumor spheroids was studied as well. As shown in Figure 6A, the fluorescence intensity of the tumor spheroid exposed to MSC-micelles was higher than that exposed to MSC-NPs. Coumarin 6 delivered by MSC-micelles penetrated into the center of the spheroids more readily. MSC-micelles with good tumor tropism can migrate and attach to C6 spheroids, with drug release and penetration concerning the transcytosis in the following drug delivery process. The antitumor activity of MSC-micelles against tumor spheroids was dose- and MSC number-dependent (Figure 6B). Up to 50% inhibition could be achieved by using 5 × 10^4^ MSC-micelles (middle-dose loading). These data suggested that MSC-micelles might be potential drug carriers in the aspect of drug delivery not only in a 2D monolayer but also in 3D tumor spheroid models.

### 2.6. Intratumoral Distribution and Antitumor Activity of MCS-Micelles

**Figure 7:**
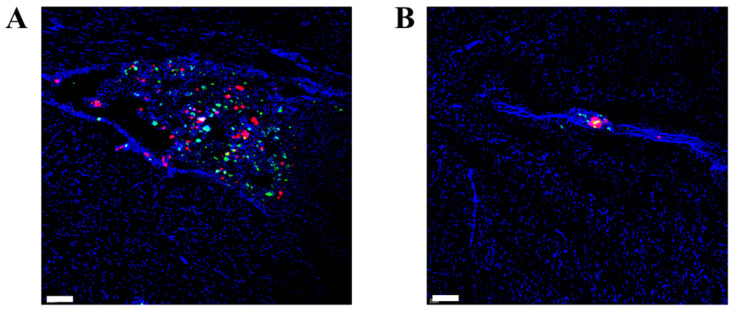
CLSM images of (**A**) glioma and (**B**) brain tissue at the injection site 2 days after contralateral injection of MSC-micelles to orthotopic C6 glioma-bearing rats. Oregon Green 488 conjugated PTX (green) was entrapped in HA-PLGA micelles, and MSCs were labeled by CM-Dil (red). The dual-labeled MSC-micelles were fabricated to track the distribution of both MSCs and PTX, simultaneously. Nuclei were stained with DAPI (blue) (bar = 100 µm).

The intratumoral distribution of MSCs and PTX after contralateral injection was tracked by the dual-labeled MSC-micelles with CM-Dil-stained MSCs and Oregon Green 488 conjugated paclitaxel. Two days after the injection into orthotopic C6 glioma-bearing rats, both MSCs (red) and fluorescent PTX (green) extensively spread throughout the tumor mass while only a few MSC-micelles (yellow dots) were scattered in the histosections shown in Figure 7A. At the injection site, the signals of MSCs and PTX were much weaker than those in the contralateral hemisphere where the glioma was located (Figure 7B). The non-colocalization of MSCs and PTX with the dominant distribution in the tumor mass suggested that MSC-micelles had excellent tumor tropism towards C6 glioma in vivo and were able to release and deliver payloads deep inside the solid tumor.

**Figure 8:**
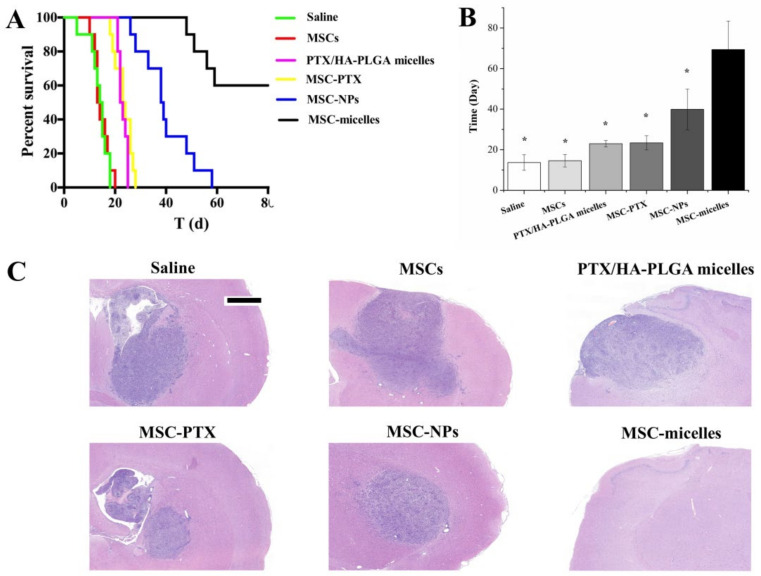
(**A**) Kaplan–Meier survival curve (*n* = 10). (**B**) Median survival time (* *p* < 0.05). (**C**) H&E sections of tumor tissues from C6 glioma-bearing rats. The samples were collected on the 7th day after therapeutic injection (bar = 1 mm).

Finally, the anti-glioma efficacy of MSC-micelles was evaluated in vivo. MSC-micelles, MSC-NPs, MSC-PTX, and PTX/HA-PLGA micelles were contralaterally injected at 1 µg PTX/kg. The therapeutic number of MSCs was controlled at approximately 2 × 10^5^. The Kaplan–Meier plot presented in Figure 8A and mean survival time calculated in Figure 8B showed a prolonged life span in PTX-formulation groups. PTX/HA-PLGA micelles and MSC-PTX had similar anti-glioma efficiency, with mean survival time being 23.0 ± 1.6 days and 23.4 ± 3.5 days, respectively. MSC-nanocarriers were superior to PTX-primed MSCs and PTX loaded micelles. It was noteworthy that MSC-micelle treatment dramatically extended the survival time of glioma-bearing rats, and only four rats died within 80 days. The remaining six rats in the MSC-micelles group were sacrificed on the 80th day without obvious abnormalities in neurological function or rat status.

H&E stained glioma images collected on the seventh day after drug injection showed a large number of tumor cells in the brain with amplified hyperchromatic nuclei from the saline and MSC groups (Figure 8C). PTX formulations induced cell destruction, extensive damaged areas with shrunken nucleus, and reduction of tumor area. The prominent activity was observed in the MSC-micelles groups. The tumor nest was invisible as a sign of glioma cure in the brain.

## 3. Discussion

Transcytosis is the rapid uptake of a ligand on one side and exocytosis on the opposite side of the cell. It enables transendothelial and transcellular drug transport, and can make the nanomedicines actively infiltrate throughout the tumor to reach the distal cells [4]. Till now, transcytosis facilitation has remained a basic challenge in nanomedicine delivery. The whole process consists of endocytosis, vesicular transport across the cell, and exocytosis of particles. Several pathways are involved and crosstalk often takes place between these pathways. Endocytosis is responsible for uptake of substances from outside the cells, while exocytosis deals with the discharge of unwanted molecules and particles from the intracellular space by fusion of the vesicular membrane with the outer cell membrane [19]. Strategies for endocytosis and cell retention enhancement in malignant cells have been extensively studied in the existing literature [22,23]. However, the phenomenon of excretion of these materials from cells is less explored because it is deemed as an unfavorable factor for drug accumulation. In the arena of cell-based drug delivery systems, not only endocytosis but also augmented exocytosis is emphasized for efficient drug loading and releasing.

In this context, we chose PLGA-derived nanocarriers to deliver the payloads between MSCs and tumor cells because PLGAs are biodegradable, nontoxic, noncumulative, and have been approved by the drug regulatory authorities of many countries. Most importantly, nanoparticles can be exocytosed out of cells in a relatively longer time than drug efflux by P-gp or exosomes [24,25,26]. PLGA NP-primed MSCs providing sustained and enhanced drug release over drug-primed MSCs were established and applied to treat orthotopic glioma in our previous study [14]. In order to achieve better therapeutic efficacy, a greater extent of intracellular drug needs to be transferred from MSCs to glioma cells.

The receptor-mediated transcytosis is a fundamental mechanism for transportation of biological nutrients and viruses. Compared with the exocytosis events of unmodified nanoparticles, the efficiency of transcytosis is significantly improved with ligand-modified nanocarriers. Therefore, various modified nanoparticles have been fabricated to facilitate the drug delivery across endothelial cells at the blood–brain barrier [27], endo- and epithelia cells at the intestinal barrier [28]. HA is an endogenous polysaccharide serving as one of the important components of the extracellular matrix. The interaction between HA and CD44 located at the MSC surface plays a significant role in several cell signal pathways that stimulate MSC adhesion, migration, homing, proliferation, and cellular differentiation [29,30,31]. CD44-mediated endocytosis of HA-PLGA micelles which we used as an HA-decorated nanocarrier was fast and remarkably enhanced in MSCs (Figure 2A), and the internalization mechanism was verified by the competition experiment.

Endocytic pathways for nanoparticulates whose size are around 200 nm include clathrin-dependent endocytosis, caveolae-dependent endocytosis, and macropinocytosis. Different endocytosis pathways lead to different intracellular trafficking fates. After clathrin-/caveolae-dependent endocytosis, particulates are enclosed in early endosomes. They are subsequently recycled in recycling endosomes or the trans-Golgi network, or transported to late endosome and finally locate in lysosomes. During these processes, Golgi apparatus excretion, fusion of caveolae or caveosomes with the plasma membrane, lysosomal fusion with the plasma membrane will occur for contents exocytosis [7,28]. HA modification has been found to assist nanocarriers to enter cells via caveolae-mediated endocytosis [32]. In this study, the reduction of micelles uptake in the presence of filipin also pointed to caveolae-mediated endocytosis of HA-PLGA micelles, while the endocytosis of PLGA NPs was not affected (Figure 3A). Designing NPs to favor caveolae-mediated endocytosis potentially enhances transcytosis [33,34]. Besides lysosomal exocytosis and Golgi apparatus excretion, HA-PLGA micelles are able to be discharged from MSCs via caveolae fusion. Therefore, more PTX was released from MSC-micelles over MSC-NPs (Figure 3C,D). Compared with MSC-PTX, both MSC-NPs and MSC-micelles showed sustained PTX release. The drug excretion closely related with P-gp efflux and particle exocytosis [9,35]. The time taken by a nanocomplex to exocytose depends on its subcellular localization and can be broadly categorized into fast (from newly formed endosomes), slow (early recycling complexes) and delayed recycling (for nanocomplexes ending up in a multivesicular body) [19]. These processes are slower than drug efflux, especially the last two, resulting in several days of cellular retention time for PLGA-derived nanoparticles (Figure 3C) [14,15].

The exocytosed nanocarriers from MSCs would infect the adjacent tumor cells after MSC migration. The intercellular delivery of coumarin 6 to C6 cells in the transwell system was enhanced by MSC-micelles (Figure 5A). Consistently, the exocytosed PTX micelles could inhibit cell viability more efficiently than PLGA NPs (Figure 5B). Due to the abundant surface markers of CD44 on C6 cells (Figure S1), we expected that the same transcytosis pattern would take place in both 3D tumor spheroids and tumor tissues. As a result, MSC-micelles possessed strong tumor penetration capacity in C6 spheroids (Figure 6A). Following a contralateral injection, MSC-micelles with good tumor tropism could home towards the tumor site relying upon their ability to interact with the cellular and chemical components of the microenvironment. Figure 7 showed that most MSCs finally located at the target site within 2 days, demonstrating that MSC-micelles could also penetrate glioma and successfully facilitate PTX to overcome tumor barriers via sequential intercellular delivery. Although the intracellular PTX level in MSC-micelles was much lower than other MSC-PTX systems [36], the anti-glioma effect was extremely potent. The relatively low PTX loading preserved the homing activity of the MSCs and enabled MSC-micelles to migrate from injection site to tumor tissue in the contralateral hemisphere (Figure 7). The discrepancies between migratory recovery time in vitro (3–5 days) and distribution time at the contralateral hemisphere in vivo (2 days) might come from the acceleration of recovery time in vivo [14]. MSC migration might take less than one day. The unreleased PTX could be further exocytosed in the form of PTX/HA-PLGA micelles by MSCs in tumors. Owing to the mechanical filtration of circulating MSCs and the impediment of the blood–brain barrier, systematic injection of therapeutic MSCs is challenging for glioma therapy. Alternatively, local administration strategies, including intratumoral, contralateral and intracerebral injections of MSCs, have been successfully applied to increase drug concentration at desired sites in preclinical studies [11,37,38]. Herein, we chose the contralateral injection for glioma therapy. However, this administration route would be unacceptable for patients. We suggest the injection of MSC-micelles into the tumor cavity after resection as the potent candidate. Further studies are needed to evaluate its efficacy and safety.

Glioma is the most common primary tumor in the brain. It causes significant mortality and morbidity [39]. Due to its highly proliferative, infiltrative and invasive properties, glioma therapy remains a challenge. As the key regulators of tumor fate, MSCs possess tumor-promoting or suppressing effects [40]. In our previous study, contralateral implantation of 2 × 10^5^ MSCs in orthotopic glioma rats were demonstrated to be safe [14]. There were no significant differences in survival time or tumor regions between those injected with unloaded MSCs and saline in Figure 8. The histological safety evaluation of MSCs at the original injection site is shown in Figure S2. We made the tissue slide right at the needle insertion position. H&E results showed relative safety of MSC-micelles, as no remarkable abnormalities in the brains were observed. This would relieve concerns about off-target toxicity. The targeted and deep delivery of PTX into tumors discussed above enabled the MSC-micelles to have the strongest antitumor activity. In present MSC-based delivery systems, median survival time was closely related to the released amount of PTX (Figure 8A,B). Although HA-PLGA PTX micelles could actively target to the cancer cells, they lack the tumor-homing capacity without the help of MSCs. Surprisingly, MSC-micelles were able to eradicate glioma, no obvious tumor nest was found in the histologic sections of the brain in six cases.

## 4. Materials and Methods

### 4.1. Chemicals and Reagents

Hyaluronic acid (Mw 5.8 kDa) was purchased from Shandong Freda Biopharmaceutical Co., Ltd. (Linyi, China). PLGA (50:50) polymer, Resomer RG 502H (Mw 12–14 kDa), 1,4-diaminobutane, sodium cyanoborohydride (NaCNBH_3_), cytochalasin D (Cyt D), filipin III, chlorpromazine (CPZ), 2-(4-fluorobenzoylamino)-benzoic acid methyl ester (Exo 1) were purchased from Sigma-Aldrich (St. Louis, MO, USA). Coumarin 6 and paclitaxel (PTX) were purchased from Aladdin Reagent (Shanghai, China). Oregon Green 488 conjugated paclitaxel (Flutax-2) and chloromethyl-1,1-dioctadecyl-3,3,3′,3′-tetramethylindocarbocyanine perchlorate (CM-Dil) were purchased from Invitrogen Life Technologies (Carlsbad, CA, USA). Cell culture media (low glucose DMEM and DMEM/F12) were purchased from Genom Pharmaceutical Biotechnology (Hangzhou, China). Fetal bovine serum (FBS) and horse serum (HS) were provided by Thermo Fisher Scientific (Waltham, MA, USA). FITC-CD44 antibody and FITC-IgG1 were purchased from Novus Biologicals (Littleton, CO, USA).

### 4.2. Cell Cultures

C6 glioma cells were purchased from the Institute of Biochemistry and Cell Biology, Shanghai Institute for Biological Science, Chinese Academy of Science (Shanghai, China). Cells were cultured in DMEM/F12 supplemented with 15% horse serum, 2.5% FBS, penicillin, and streptomycin.

Bone marrow-derived MSCs were isolated from the bone shaft of femurs of 3-week-old male Sprague–Dawley rats (Shanghai Slac Laboratory Animal Co., Ltd., Shanghai, China) as per our previously reported method [14]. MSCs were cultured with low-sugar DMEM containing 10% FBS. Second- to fifth-passage cells were used for the experiment.

### 4.3. Synthesis and Characterization of HA-PLGA

HA-PLGA block copolymer was synthesized by an end-to-end coupling strategy with amino-functionalized HA and N-Hydroxysuccinimide PLGA (PLGA-NHS) as described in references [18,41]. Briefly, diaminobutane with HA dissolved in H_2_O were stirred at 50 °C for 24 h. Then, sodium cyanoborohydride was added to the mixture and the amino-functionalized HA was purified by dialysis. PLGA-NHS was synthesized by dissolving PLGA-COOH, NHS, and EDC·HCl in methylene chloride, and stirred for another 24 h. Finally, PLGA-NHS and amino-functionalized HA with N, N-Diisopropylethylamine were added into DMSO with stirring at 50 °C for 48 h. HA-PLGA was purified and dried by dialysis and lyophilization, respectively.

Molecular weight of HA-PLGA was determined by gel permeation chromatography (GPC) (Waters Breeze, Milford, MA, USA).

### 4.4. Preparation and Characterization of PTX/HA-PLGA Micelles

PTX/HA-PLGA micelles were prepared by the modified solvent-dialysis method. Briefly, 10 mg HA-PLGA with 0.5 mg PTX were dissolved in 1 mL DMSO/DMF (*v*/*v* =3:1) mixture. Then, 4 mL deionized water was slowly added under stirring, and PTX/HA-PLGA micelles were developed for 10 min. Extra organic solvent was removed by dialysis (Spectra/Po, MWCO: 3.5 kDa) against deionized water for 12 h. Unencapsulated PTX was removed by centrifugation at 3000 rpm for 10 min and filtered through a syringe filter (0.8 µm, Millipore, Billerica, MA, USA). PTX-loaded PLGA nanoparticles (PTX/PLGA NPs) were set as the control particles which were prepared by the modified emulsion solvent evaporation method [12]. Then, 5 mg PTX and 100 mg PLGA were added to 5 mL CH_2_Cl_2_, then 25 mL deionized water with 1% polyvinyl alcohol was added. The mixture was sonicated and stirred to evaporate CH_2_Cl_2_. Fluorescent particles loaded with coumarin 6 and Flutax-2 were prepared in the same way as PTX-loaded micelles and nanoparticles, respectively.

Transmission electron microscopy (JEM-1230, JEOL, Tokyo, Japan) was used to observe the morphology of PTX/HA-PLG micelles. Particle size and zeta potential were determined by laser light scattering (Zetasizer Nano ZS, Malvern, UK). In vitro release of PTX/HA-PLGA micelles was investigated using dialysis against 0.1% Tween 80 in PBS (pH 7.4). Dialysate was collected and analyzed by HPLC (Alliance 2690; Waters, Milford, MA, USA). Detection conditions were: Diamonsil C_18_ column (250 × 4.6 mm, 5 μm); flow rate 1 mL/min, wavelength 227 nm. The mobile phase was acetonitrile:water (55:45).

### 4.5. Cell Uptake

Cell uptake was evaluated by CLSM (FV1000, Olympus, Tokyo, Japan). 500 μL medium containing (I) coumarin 6/PLGA nanoparticles; (II) coumarin 6/HA-PLGA micelles; (III) coumarin 6/HA-PLGA micelles with free HA (5 mg/mL) which had a final concentration of coumarin 6 (200 ng/mL) were added to 3 × 10^4^ MSCs for 8 h. The cells were fixed with 4% formaldehyde and stained by DAPI. Uptake by C6 cells was evaluated in a transwell system (8 µm pore size; Costar, Corning, NY, USA). After incubation with coumarin 6/HA-PLGA micelles (500 ng/mL) for 8 h, 3 × 10^4^ MSC-micelles were trypsinized and seeded onto the upper chamber while 1 × 10^5^ C6 cells were added in the lower one. C6 cells were fixed and observed 24 h later.

### 4.6. Treatment with Endocytosis/Exocytosis Inhibitors

MSCs were pre-incubated for 30 min with three endocytosis inhibitors: CPZ (10 µg/mL), Cyt D (1 µg/mL), and filipin III (1 µg/mL), respectively. Then, coumarin 6/PLGA NPs and coumarin 6/HA-PLGA micelles (0.5 µg/mL) were added to cells for 8 h. Cells were washed with PBS and lysed. In order to find out whether the cellular uptake was energy dependent, cells were incubated at 4 °C. The results were reported as mean fluorescence intensity of cells with inhibitors normalized to the cells without inhibition treatment.

For the exocytosis study, MSCs incubated with coumarin 6/PLGA NPs or coumarin 6/HA-PLGA micelles (0.5 µg/mL) for 4 h were washed and the medium was replaced with fresh medium. Exo 1 and Cyt D were added with the final concentration of 50 µM and 10 µg/mL, respectively. After 2 h, the medium was removed and cell lysate was collected for fluorescence determination. The fluorescence intensity of cell lysate was measured at λex = 456 nm, λem = 504 nm. The results were reported as mean fluorescence intensity of cells with inhibitors and normalized relatively to the cells without inhibitors as control.

### 4.7. Cytotoxicity of MSC-Micelles

The cytotoxicity of PTX/HA-PLGA micelles and MSC-micelles were determined by MTT assay. PTX/HA-PLGA micelles, PTX/PLGA NPs, and PTX solution ranging from 0.5 to 15,000 ng/mL or 1 to 100 ng/mL were incubate with MSCs (5 × 10^3^ cells) and C6 cells (1 × 10^4^ cells) for 72 h, respectively. Then, cells were washed and treated with MTT solution for 4 h. The absorbance of crystals dissolved by DMSO was determined at 570 nm by a microplate reader (BioTek, Winooski, VT, USA).

In vitro antitumor effect of MSC-micelles and MSC-NPs against C6 cells was evaluated by a transwell system. MSC-micelles and MSC-NPs were constructed by incubating MSCs with 5–10 ng/mL PTX/HA-PLGA micelles or PTX/PLGA NPs for 8 h, respectively. Then, MSC-micelles and MSC-NPs were trypsinized and seeded on the upper chamber, while C6 cells were added in the lower one at the cell number ratio of 1:5, 1:3, and 1:1, respectively (MSC-nanocarriers/C6). The viability of C6 cells was analyzed by MTT assay 3 days later.

### 4.8. Characterization of MSC-Micelles

The tumor tropism of MSC-micelles in vitro was determined by a migration assay where 3 × 10^4^ MSC-micelles (incubating MSCs with 5, 8, 10 ng/mL PTX/HA-PLGA micelles for 8 h) were seeded in the upper chamber and 1 × 10^5^ C6 cells were placed into the lower well. The cells that did not migrate through the pores were removed with cotton swabs 1 d, 3 d, and 5 d after seeding, respectively. The migrated ones on the lower surface of the membrane were fixed with methanol and stained with 0.1% crystal violet. The MSCs without micelle treatment were set as the control. The number of stained cells was counted and the migration rate was calculated: counts (sample)/counts (control) × 100%.

Cell cycle of MSC-micelles was detected by flow cytometry. MSC-micelles constructed as described above were cultured with PTX-free medium. Then, 0 d, 1 d, 3 d and 5 d later, cells were fixed and stained with propidium iodide (PI)/RNase. Fluorescence intensity was analyzed in the FL-3 channel.

### 4.9. Drug Release Kinetics and Transfer from MSC-Micelles to C6 Cells

MSCs (10^6^ cells) were treated with PTX/HA-PLGA micelles, PTX/PLGA NPs and PTX solution for 8 h (8 ng/mL), respectively. Then, cells were washed and incubated with drug-free medium. At predetermined time points, the cells were collected and mixed with methanol. After sonication, the samples were centrifuged and the supernatant was air-dried, redissolved in methanol. Intracellular PTX level was analyzed by HPLC.

To investigate the drug transfer from MSC-nanocarriers to C6 cells, coumarin 6 was used as a fluorescence probe. MSCs were incubated with coumarin 6/HA-PLGA micelles or coumarin 6/PLGA NPs (500 ng/mL, coumarin 6-equiv.) for 4 h, respectively. Then 3 × 10^4^ MSCs-nanocarriers were collected and seeded in the upper chamber of the transwell system while the lower well was seeded with 1 × 10^5^ C6 cells. After co-culturing for 24 h, the tumor cells were fixed with 4% paraformaldehyde for 30 min and stained with DAPI before CLSM observation.

### 4.10. Tumor Spheroid Penetration and Viability

C6 tumor spheroids were developed using a liquid overlay system. Briefly, 1 × 10^5^ C6 cells were seeded into a 24-well plate which was pre-coated with the layer of agarose. The round, dense and well-organized tumor spheroids were selected for the further studies 10 days later.

Coumarin 6/HA-PLGA micelles and coumarin 6/PLGA NPs (500 ng/mL, coumarin 6-equiv.) were incubated with MSCs for 4 h, respectively to fabricate fluorescent MSC-nanocarriers. Then, 1 × 10^5^ MSC-nanocarriers were co-cultured with tumor spheroids. After incubation for 48 h, tumor spheroids were washed and fixed with 4% paraformaldehyde for CLSM observation. The spheroid viability was determined by trypan blue assay. 1 × 10^4^, 5 × 10^4^, and 1 × 10^5^ MSCs-nanocarriers (5–10 ng/mL, PTX equiv.) were added to C6 tumor spheroids. After 48 h incubation, sets of spheroids (10 per set) were collected and trypsinized with 0.25% trypsin-EDTA. Only dead cells could be stained with 0.4% trypan blue. The number of positive cells and total cells were determined on a hemocytometer.

### 4.11. Intratumoral Distribution of MSC-Micelles

An orthotopic brain tumor model was performed according to our previous work [14]. Briefly, 10^6^ C6 glioma cells were injected into the left forebrain (3 mm lateral, 1 mm anterior to bregma, 5 mm depth from the skull surface) of rats. Seven days later, 2 × 10^5^ dual labeled MSC-micelles (incubating CM-Dil stained MSCs with 8 ng/mL PTX-Flu/HA-PLGA for 8 h) were contralaterally injected into the right forebrain (3 mm lateral, 1 mm anterior to bregma, 5 mm depth from the skull surface). Frozen specimens of brain tumor and contralateral tissue were collected two days later and observed by CLSM. The relative locations for C6 implantation, MSC-micelles injection, and CLSM observation are shown in Figure S3.

### 4.12. Antitumor Effect In Vivo

Seven days after tumor inoculation, the rats were randomly divided to 5 groups (*n* = 10) receiving a contralateral injection at the dose of 1 µg PTX-equiv./kg, of (a) saline; (b) PTX/HA-PLGA micelles; (c) MSCs (2 × 10^5^ cells); (d) MSCs-PTX (2.2 × 10^5^ cells); (e) MSC-NPs (2 × 10^5^ cells); (f) MSC-micelles (1.8 × 10^5^ cells). The injection volume was 10 µL. Survival time was recorded and the rats were sacrificed when they had lost more than 15% body weight. Hematoxylin and eosin staining (H&E) was utilized for the histopathology evaluation 7 days after therapeutic injection.

### 4.13. Statistical Analysis

Data were shown as mean ± SD. Statistically significant differences were analyzed by one-way ANOVA followed by SNK or Dunnett-t test. The probability of survival was estimated by the Kaplan–Meier method and compared by the log-rank test in GraphPad Prism 6.0. Statistical significance was defined by * *p* < 0.05.

## 5. Conclusions

An MSC-based drug delivery system with tumor tropism and penetration was successfully established for glioma therapy. By taking advantage of over-expressed CD44 on both MSCs and C6 cells, effective drug loading, improved drug transfer through transcytosis, deep penetration and preferable drug accumulation in tumors were achieved. The MSC-micelles exhibited a strong anti-glioma effect providing a promising strategy for tumor chemotherapy.

## Data Availability

This data presented in this study are available in the Supplementary Materials.

## Supplementary Materials

The following are available online at https://www.mdpi.com/article/10.3390/molecules27082419/s1, Figure S1: Flow cytometric analysis of CD44 expressed on the surface of (A) MSCs and (B) C6 glioma cells. Figure S2: H&E staining of brain section at the MSC-micelles injection site. Figure S3: The relative locations for glioma implantation, MSC-micelles injection, and CLSM observation (cross section).

## Sample Availability

Samples of the compounds are not available from the authors.
