# Supplementary material for: Tumor Tropic Delivery of Hyaluronic Acid-Poly (D,L-lactide-co-glycolide) Polymeric Micelles Using Mesenchymal Stem Cells for Glioma Therapy
Source: Molecules. 2022 Apr 8;27(8):2419. doi: 10.3390/molecules27082419 (PMC9027425; doi:10.3390/molecules27082419)
Supplement: Supplementary file 1 [file molecules-27-02419-s001.zip › molecules-1660632-supplementary.pdf]

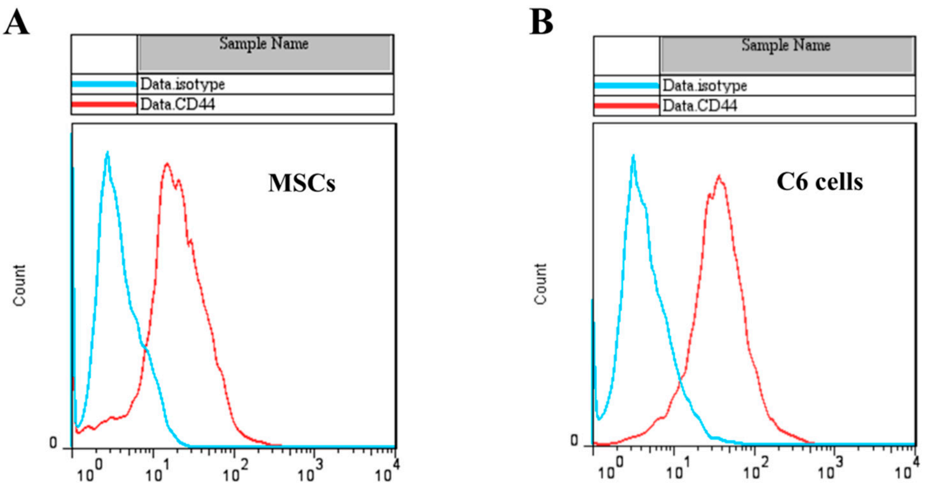

Figure S1: Flow cytometric analysis of CD44 expressed on the surface of (A) MSCs and (B) C6 glioma cells.

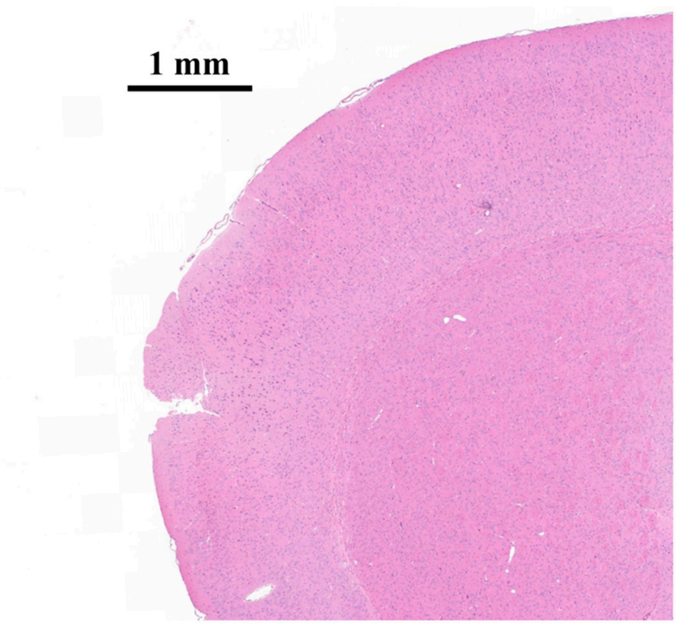

Figure S2: H&E staining of brain section at the MSCs-micelles injection site.

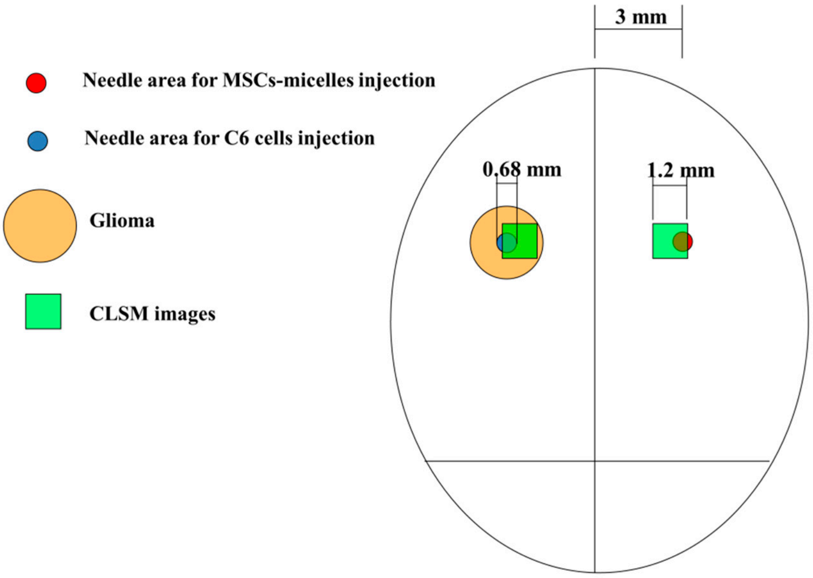

Figure S3: The relative locations for glioma implantation, MSCs-micelles injection, and CLSM observation (cross section).
